# Enantioselectivity Induced by Oxazaborolidine Supported on Mesoporous Silica or by Its Analog in Homogeneous Phase

**DOI:** 10.3390/molecules15053643

**Published:** 2010-05-18

**Authors:** Jeremy H. Yune, Françoise Quignard, Karine Molvinger

**Affiliations:** Institut Charles Gerhardt Montpellier, UMR 5253 CNRS/ENSCM/UM2/UM1, Matériaux Avancés pour la Catalyse et la Santé, « MACS » - 8, rue de l’Ecole Normale, 34296 Montpellier cedex 5, France

**Keywords:** oxazaborolidine, supported catalyst, mesoporous silica, asymmetric reduction of acetophenone

## Abstract

The impact of immobilization of oxazaborolidines supported on silica via different substituents on the boron and nitrogen atoms is evaluated in the enantioselective reduction of acetophenone. The performances of the homogeneous analog oxazaborolidines and silica-supported ones are compared by varying different parameters. This article deals with the synthesis, characterization and catalytic evaluation of silica-supported oxazaborolidines, their recycling capabilities and regeneration limitations.

## 1. Introduction

**Figure 1:**
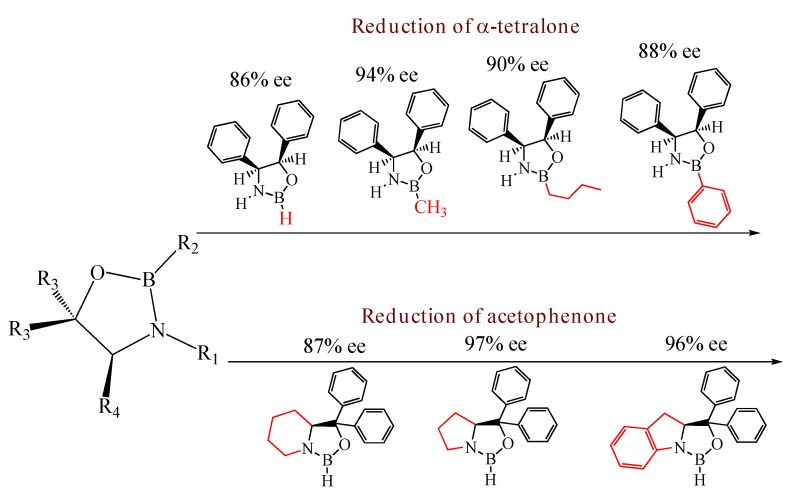
Influence of substituent linked to oxazaborolidine boron and nitrogen element on the enantioselectivity.

Since their discovery by Itsuno *et al.* in 1981 [1] and their major development by Corey *et al.*, chiral 1,3,2-oxazaborolidines have proven to be highly effective homogeneous catalysts for the enantioselective reduction of ketones to chiral secondary alcohols by borane. The mechanism has been elucidated by Corey *et al*. [2]: in fact the oxazaborolidine (synthesized from an amino alcohol) forms a complex with the borane and this complex rapidly reduces the ketones with excellent yields and enantiomeric excesses. Excellent results have been reported by Quallich using (1*S*,2*R*)-2-amino-1,2-diphenylethanol, since one face of the catalyst is shielded due the orthogonal arrangement of the phenyl substituents [3]. Furthermore, both enantiomers of Quallich’s amino alcohol are available and not very costly in comparison with Corey’s amino alcohol. Under homogeneous conditions, the enantioselective properties of oxazaborolidines are structure dependant [2], therefore the nature and the position of the substituent strongly affect the enantiomeric excess achieved (Figure 1). 

**Figure 2:**
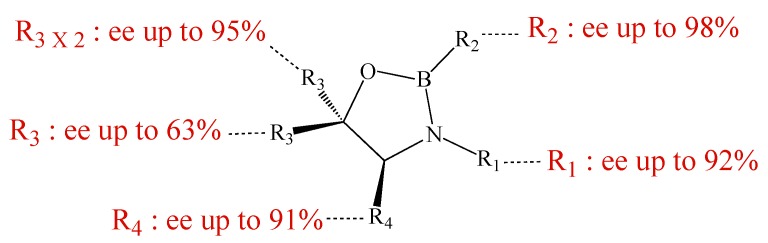
Supported oxazaborolidines by different substituents on polymer.

Since separation of the alcohol product from the chiral amino alcohol (catalyst precursor) can be difficult [4], metal [5,6] or polymer [7,8,9,10,11,12,13,14] supported oxazaborolidines have been considered. The immobilisation was achieved through a covalent bond between the support and one of the substituents of the oxazaborolidine. Therefore under heterogeneous conditions the substituent chosen to anchor the enantioselective inductor also influences the enantioselective properties [10,11,12,13,14] (Figure 2).

In the case of a polymer supported oxazaborolidine, the ketone must diffuse into the polymer, and the solvent used has to swell the crosslinked polymer beads in order to increase accessibility to the catalytic sites. After reduction and hydrolysis, the product removal from the polymeric matrix can be difficult. In the case of metal-supported oxazaborolidines, these solids have a rather low surface area and they are not easy to manipulate. In view of these drawbacks, mesoporous silica appears as an interesting alternative due to its high surface area and mechanical resistance. In this paper, we will compare the results for the enantioselective reduction of acetophenone for different silica-supported oxazaborolidines, anchored via substituents of the boron or nitrogen element. The structural modifications induced by the procedure chosen to graft the oxazaborolidine were simulated under homogeneous conditions, and allow a direct evaluation of the homogeneous *versus* immobilized oxazaborolidine.

## 2. Results and Discussion

### 2.1. Immobilization of oxazaborolidines via boron substituent

**Figure 3:**
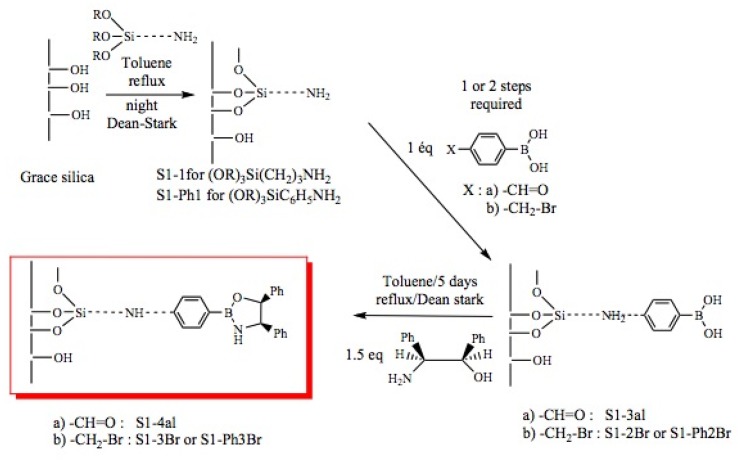
Immobilization of oxazaborolidine via boron function.

**Figure 4:**
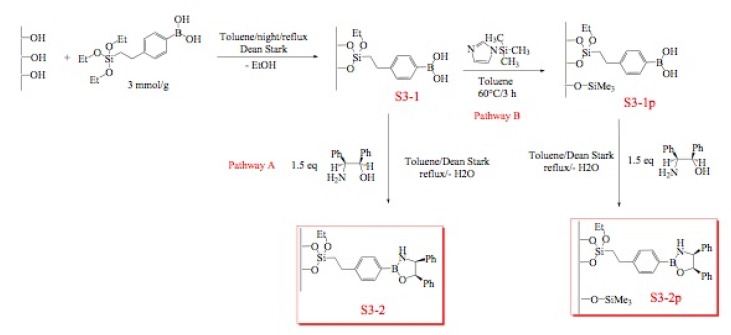
Direct immobilization of oxazaborolidine via boron function.

Immobilization of oxazaborolidines via the boron substituent is obtained by a multi-step synthesis, starting with the functionalization of the mesoporous silica by an amino alkoxysilane (aminopropyl- : S1-1 or aminophenyl- alkoxysilane S1-Ph1) then immobilization of boronic acid and finally synthesis of the chiral ligand by adding a chiral amino alcohol [(1*S*,2*R*)-diphenylaminoethanol, Figure 3). In an alternative route, the oxazaborolidine has been immobilized by a direct functionalization of silica with a boronic acid alkoxysilane (Figure 4). 

### 2.2. Characterization of the materials

Qualitative and quantitative characterizations of the immobilized species for each step of the materials synthesis are obtained by DRIFT and elemental analysis, respectively. Furthermore, the textural characterization is realized by nitrogen sorption. 

The formation of the grafted species for each step of the synthesis was monitored by DRIFT. All the spectra are standardised according to Si-O-Si overtone at 1,987 and 1,870 cm^-1^. Thus, the functionalization of silica with APTES has been confirmed by both the decrease of the intensity of the vibration bands of the isolated silanols at 3,747 cm^-1^ and the apparition of the bands at 3,378, 3,317 and 2,931, 2,867 cm^-1^ characteristic of ν(NH_2_) st and ν(C_al_-H), respectively, for S1-1. Then, the presence of ν (BO-H) st (3,300, 3,262 cm^-1^), the enhancement of ν(C_ar_-H) (3,070, 3,028 cm^-1^) for S1-Ph2Br and ν(C_al_-H) (2,931, 2,867 cm^-1^) for S1-2Br concur to confirm the grafting of boronic acids. Finally, the formation of the oxazaborolidine has been revealed by the presence of ν(B-N) st at 1,452 cm^-1^ and the δ(N-H) linked to the bore (1,650 cm^-1^) and by the increase of the ν(C_ar_-H) st and ν(C_al_-H) st (S1-3Br and S1-Ph3Br). 

The composition of each solid is determined by elemental analysis, and as the successive reactions are not quantitative, the final solid is functionalized by several species. In order to optimize the loading of the chiral ligand on silica, several parameters have been changed such as reaction time, nature of the boronic acid, amino alkoxysilane and strategy of immobilization. 

**Figure 5:**
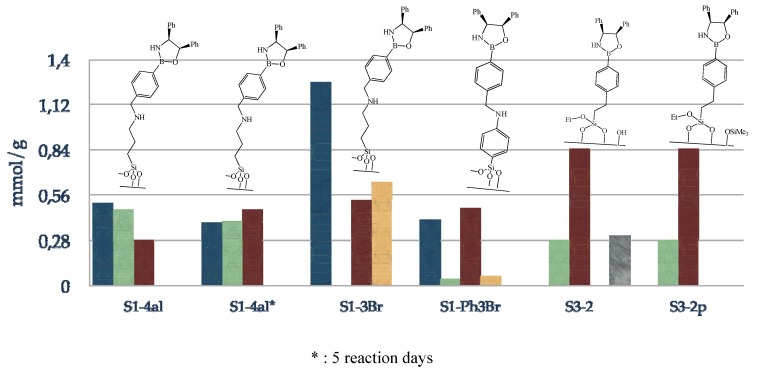
Composition of supported oxazaborolidine via boron substituent; blue: amino species, green: boronic acid species, red: immobilized oxazaborolidine, grey: physisorbed amino alcohol.

Figure 5 presents the composition of the final materials; blue, green, red and grey plots correspond respectively to amino, boronic acid, oxazaborolidine grafted and physisorbed amino alcohol. Increasing reaction time from 24 h to 5 days results in a higher loading of oxazaborolidine supported on silica, *i.e.*, 0.28 to 0.47 mmol/g. The use of 3-bromomethylphenyl boronic acid allows the immobilization of the chiral ligand in three instead of four steps with a slight improvement of the chiral ligand loading, 0.53 mmol/g is obtained via this method. S1-Ph3Br has been synthesized in order to determine the influence of the grafted species rigidity on enantioselectivity since a phenyl substituent linked to the surface is known to be less flexible than an alkyl substituent. Finally, immobilization of the chiral ligand via a direct functionalization of silica with boronic acid alkoxysilane is the most appropriate method to obtain the highest chiral ligand loading, 0.85 mmol/g, moreover in fewer steps. Physisorption of the amino alcohol can be avoided by an intermediate passivation step.

### 2.3. Immobilization of oxazaborolidines via nitrogen substituent

**Figure 6:**
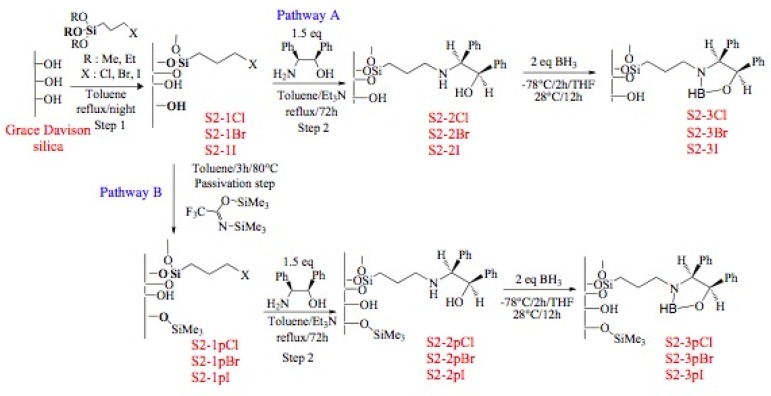
Immobilization of oxazaborolidine via nitrogen substituent.

Immobilization of oxazaborolidines via the nitrogen substituent is achieved by a multi-step synthesis (Figure 6, pathway A), the first one required the functionalization of the mesoporous silica by co-condensation of the surface silanols with an alkoxysilane presenting an halogen function. Several halogens (Cl, Br, I) have been used in order to compare their reactivity in the nucleophilic substitution reaction. The chiral amino alcohol has been supported thanks to a nucleophilic substitution between amino and the halogen functions grafted on the mesoporous silica. Finally, the oxazaborolidine has been synthesized by addition of borane according to classical homogeneous phase procedures.

In order to study the influence of residual silanols on conversion of the acetophenone, a passivation step has been realized (Figure 6, pathway B).

### 2.4. Characterization of the materials

Regarding the synthesis for S2-2X and S2-2pX, the functionalization of the silica by the halogenopropyltriethoxysilane has been confirmed by the decrease of the intensity of the vibration bands of the isolated silanols at 3,747 cm^-1^ and the apparition of the bands at 2,931 and 2,867 cm^-1^ characteristic of ν_as_(C_al_-H) and ν_s_(C_al_-H), respectively. Then, immobilization of the chiral amino alcohol converts a secondary into primary amino alcohol; this procedure is evidenced by the presence of the bands at 3,070, 3,028 cm^-1^ and 3,340 cm^-1 ^characteristic of ν(C_ar_-H) and ν(N-H) st, respectively and the increase of ν(C_al_-H). Although DRIFT is not quantitative, we can observe that in the case of the Cl grafted on silica, less amino alcohol reacts due to the less nucleophile character of the Cl compared to Br and I. The passivation step is confirmed by the decrease of the vibration bands of the isolated silanols at 3,747 cm^-1^. A lower amount of amino alcohol grafted on S2-2pI compared to S2-2I is expected since the vibration bands intensity of ν(C_ar_-H) and N-H st are lower.

**Figure 7:**
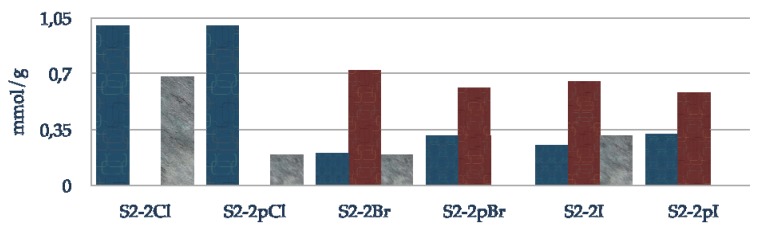
Composition of supported oxazaborolidine through nitrogen substituent; blue: halogeno species, red: chemisorbed amino alcohol, grey: physisorbed amino alcohol.

Figure 7 describes the composition of the final material with oxazaborolidine supported via the nitrogen substituent. The blue plots represent the loading of efficiently grafted halogeno fragment, the red and grey plots correspond to grafted and physisorbed amino alcohol, respectively. To reach high loadings of immobilized amino alcohol, it is preferable to use iodo- and bromo- rather than chloro-alkoxysilane. Passivation of residual silanols prevents the physisorption of the amino alcohol. Nevertheless, the reactivity seems to be altered since the loading of grafted amino alcohol is lower with each alkoxysilane due to the hydrophobisation of the surface. Effectively, as observed in DRIFT, no amino alcohol is grafted in the case of the chloro propyl functionalization, it is just physisorbed.

### 2.5. Textural properties of the materials

The textural properties of the materials have been obtained by nitrogen sorption experiments and all the isotherms have been standardised according to the inorganic part. For both pathway, pores volume and diameter decrease after each step as the size of the organic species grafted on the silica increases, which prove immobilization of species inside the pore. C parameter attests the surface polarity and diminishes according to the augmentation of organic species. As expected, this parameter is lower for the material S2-1pI, where a passivation step has been realized. Furthermore, the nature of the halogen does not affect the texture of the materials. In Table 1, the textural properties of the supported oxazaborolidines which are used in catalysis are reported.

**Table 1:** Textural properties of supported oxazaborolidines.

Samples	Surface area (m^2^.g^-1^)	V_pores_ (cm^3^.g^-1^)	Pore diameter (Ǻ)	C
SG	523	1.93	160	105
S2-3Br	457	1.56	98	40
S2-3pBr	390	1.40	98	29
S2-3dI	423	1.47	100	43
S1-3Br	382	1.27	93	43
S1-Ph3Br	455	1.50	92	47
S3-2	346	1.11	93	55
S3-2p	333	1.17	96	27

### 2.6. Catalytic results

The final solid is functionalized by several species, and in order to check the influence of these residual species on the silica, the reduction of acetophenone by BH_3_ has been realized in the presence of various samples presenting a precise surface environment (Table 2). The reaction involves 0.7 equivalents of borane in THF with acetophenone to achieve a complete conversion since the borane provides two hydrides and thus can reduce two equivalents of ketone [15,16] (entry 1). Borane (BH_3_ Lewis acid) can coordinate to an OH function (Lewis base), resulting in its loss of activity, as previously observed by Asefa *et al.* [17]. The comparison of entries 2 and 3 proves without ambiguity the negative influence of the silanol on conversion. Since the passivation of all the silanols cannot be completed, some residual silanols are still available, leading to an incomplete conversion. Through coordination between amino, halogen and borane, formation of halogenoborane species can occur [18]. As a consequence, conversion is not complete in the presence of S2-1I and S1-1 (entries 4 and 6). According to entry 5, a lower conversion compared to entry 2 is obtained, which can be the result of the potential coordination of the borane on both residual silanol and physisorbed amino alcohol. Since this physisorbed species can form a chiral ligand by the addition of borane during the catalytic reaction, two reduction cycles have been realized in order to evaluate its influence on enantiomeric excess. No chiral induction has been observed during the reduction of acetophenone for both cycles, evidencing the fact than the potential oxazaborolidine physisorbed cannot induce chirality in this precise environmental configuration. Indeed, silanol could interact with the endocyclic boron of the ligand leading to the impediment of the required complexation of the ketone. Boronic acid species also damage the conversion due to the complexation of borane on OH groups of the boronic acid mentioned by Caze *et al. *and Martens *et al.* [19,20] and confirmed by the comparison between entries 6 and 7.

As a consequence, activities of the supported oxazaborolidine are expected to be lower than the homogeneous analog but none of the residual fragments will act as a chiral inductor. Thus the enantioselectivity will be directly coupled with the presence of immobilized oxazaborolidine.

**Table 2:** Influence of residual species on conversion.

Entry	Sample	Conversion (%)*	ee (%)
1	BH_3_^1^	**100**	0
2	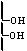	**67**	0
3	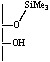	**89**	0
	Grace Davison silica end-capped		
4	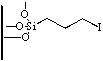	**70**	0
	S2-1I		
5	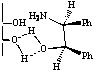	**40**	0
		**61 ^2^**	0
6	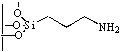	**77**	0
	S1-1		
7	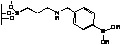	**52**	0
	S1-2Br		

* Selectivity of 100%; reaction time: 2 h; ^1 ^Ratio surface species/BH_3_/ketone: 0/0.7/1; ^2^ 2nd cycle.

As previously mentioned, under homogeneous conditions, the enantioselective properties of oxazaborolidines are structure dependant [2] and the nature and the position of the substituent strongly affect the enantiomeric excess achieved. Grafting the oxazaborolidine leads *de facto *to the modification of the boron or nitrogen atom substituents. Thus, the enantiomeric excess differences observed can be induced by the immobilization, rather by these substituent modifications. In order to discriminate between these two hypotheses, the same structural modifications have been introduced on soluble oxazaborolidines. Comparing the results in Table 3 from entries 1 to 4, as expected, the bulkier the substituent is, the lower the ee are, from H linked to the boron (93% ee) to triethoxy(phenylethyl)silane (80%). The oxazaborolidine (entry 4) can be considered as the homogeneous analog of the heteregeneous catalyst; in this case, the ee is only moderate.

**Table 3:** Influence of the substituent linked to the boron on the ee.

Entry	Catalyst	ee (%)
1	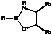	**93**
2	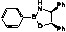	**76**
3	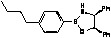	**80**
4	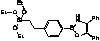	**80**

Ratio oxazaborolidine/BH_3_/acetophenone = 0.1/0.7/1; Reaction time: 1 h; Conversion: 100%.

When oxazaborolidine is immobilized on silica via the nitrogen atom, the decrease of the reactivity of complex ligand.borane toward borane can affect its enantioselectivity. The influence of a propyl group has been evaluated in homogenous phase. The enantioselectivity decreases dramatically when the hydrogen is replaced by a propyl function (Table 4). In this latter case, the steric hindrance of the propyl group blocks the complexation of borane on the nitrogen atom, the reduction by the borane alone is favored, and the enantiomeric excess (ee) diminishes. Moderate ee can be achieved by increasing the amount of oxazaborolidine used. These observations are in agreement with those reported by Quallich in the case of the reduction of tetralone [3]. He reported that the substitution of a hydrogen atom by a methyl group on nitrogen leads to a dramatic drop of the ee (94% to 0%). By using a stoechiometric ratio, 30% of ee is reached. In our case, increasing the ratio until one equivalent leads to an improvement of the ee, but is still low (45%). 

**Table 4:** Influence of the nitrogen substituent of the oxazaborolidine on the enantioselectivity.

Entry	Oxazaborolidine	Ratio Oxazaborolidine/BH_3_/ketone	Time (hours)	conversion (%)*	ee (%)
1	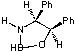	0.1/0.7/1	1	100	93
2	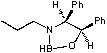	0.1/0.7/1	1	86	10
			2	100	10
		0,3/0.7/1	1	71	45
			2	100	45
		1/0.7/1	1	47	45
			2	100	45

Whatever the strategy used, the enantiomeric excesses obtained with supported oxazaborolidine are higher than those obtained in homogenous phase (Table 5, entries 1 and 2, Table 5, entries 3 and 4). Total conversion cannot be reached because of the presence of residual species on the silica, but the enantioselectivity is improved. 

**Table 5:** Enantioselectivity comparison between homogeneous and supported oxazaborolidine.

Entry	Oxazaborolidine	Ratio Oxazaborolidine/BH_3_/cétone	conversion (%)	ee (%)
1	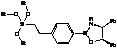	0.1/0.7/1	100	80
		1/0.7/1	100	84
2	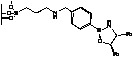	0.1/0.7/1	57	80
		0.16/0.7/1	50	81
		0.3/0.7/1	40	94
	S1-3Br			
3	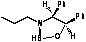	0.1/0.7/1	100	10
		0.3/0.7/1	100	45
		1/0.7/1	100	45
4	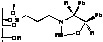	0.1/0.7/1	84	35
		0.3/0.7/1	72	72
	S2-3Br*			

Selectivity: 100%.

In homogeneous phase, the optimal temperature to obtain the highest enantioselectivity depends on the solvent, reductant and oxazaborolidine used [21,22]. Moreover, this defined temperature is not necessary equivalent when the chiral ligand is immobilized on polymers due to their swelling. The influence of this parameter has been realized with the silica-immobilized oxazaborolidine. In both homogeneous and heterogeneous phase, the influence of the temperature on enantioselectivity is similar and the best enantioselectivity is obtained at 28 ºC (Table 6). 

**Table 6:** Influence of the temperature on the enantioselectivity.

Temperature of reaction (ºC)	Oxazaborolidine	time (h)	conversion (%)	ee (%)
0	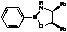	1	100	60
28		1	100	80
60	(4*S*,5*R*)-2,4,5-triphenyl-1,3,2-oxazaborolidine	1	100	56
0	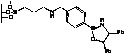	2	53 (56)	75
28		2	40 (42)	94
60	S1-3Br	2	54 (54)	72

() results after 24 h; Selectivity: 100%; oxazaborolidine/BH_3_/ketone ratio: 0.3/0.7/1.

According to the nature of the solvent used [23,24], oxazaborolidines can exist in homogeneous phase as dimers. As a matter of fact, B-N interaction between two ligands inhibits its complexation with the ketone leading to a loss of activity and consequently of ee. With the heterogeneous form, the dilution of the active site can be reached by anchoring iodopropyltrimethoxysilane and propyltrimethoxysilane (1:2 ratio) on the surface. The enantioselectivity remains the same (Table 7, entry 3), but a longer reaction time is necessary to reach high conversion. An improvement of the conversion is achieved by the attenuation of residual silanol after a passivation step from 72% to 82% (Table 7, entry 2). A better isolation of the active sites accomplished by its dilution does not improve the enantioselectivity of the final supported chiral ligand (Table 7, entry 3). It appears that immobilization is sufficient to avoid the dimerization form of oxazaborolidine.

**Table 7:** Quantification of surface species and catalytic results for the supported oxazaborolidine at 28 ºC.

Entry	Oxazaborolidine supported	Quantity of ligand(mmol/g)	conversion (%)*	ee (%)
1	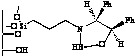	0.61	72	**72**
	S2-3Br			
2	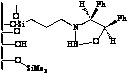	0.58	82	**72**
	S2-3pBr			
3	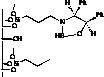	0.36	33	**72**
	S2-3dI			

Oxazaborolidine/BH_3_/ketone ratio = 0.3/0.7/1; Reaction time: 2 h.

With immobilized oxazaborolidine, diffusion limitations can affect the catalytic behaviour. Diffusion limitation can result from some intrinsic textural properties of the support, like pore size, or from the textural modifications induced by the surface functionalisation. Thus, two different kinds of experiments have been realized. In the first case, oxazaborolidine supported on silica with pore diameter at least two-fold smaller leads to a decrease of the enantioselectivity, 80% *vs.* 65% (Table 8). In the second case S1-Ph3Br presents a bulkier substituent than S1-3Br (structure in Figure 5) which may limit the accessibility to the pore. As a result, minor enantiomeric excess is obtained with S1-Ph3Br, 68% *vs.* 94% (Table 9, entries 1,2). As a consequence, an increased amount of the chiral inductor ratio (to 0.3/0.7/1) is necessary to compensate for the diffusion limitations. 

**Table 8:** Influence of the silica’s structural properties on enantioselectivity.

Entry	Oxazaborolidine supported	Surface area (m^2^/g)	Pore volume (cm^3^/g)	Pore diameter (nm)	ee (%)
1	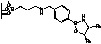	284	0.41	4.4	**65**
	S1-4				
2	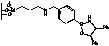	382	1.27	9.3	**80**
	S1-3Br				

Oxazaborolidine/BH_3_/ketone ratio: 0.1/0.7/1.

The recycling of the solids was studied (Table 9). Six runs were conducted with the catalyst reactivated under vacuum before re-use. The results are really convincing: the enantiomeric excess are maintained for the six runs, whatever the solid used. Moreover, conversion increases after each cycle due to the passivation of the residual surface species by the borane (BH_3_) which can potentially be coordinated to these functions. In addition, leaching of the chiral ligand has been evaluated by GC/MS, and the presence of oxazaborolidine in solution was never detected.

**Table 9:** Recycling of supported oxazaborolidine, ratio: 0.3/0.7/1(time: 2 h).

Entry	Supported oxazaborolidine	Cycle 1		Cycle 2		Cycle 3		Cycle 4		Cycle 5		Cycle 6	
		ee	Conv	ee	Conv	ee	Conv	ee	Conv	ee	Conv	ee	Conv
		(%)	(%)	(%)	(%)	(%)	(%)	(%)	(%)	(%)	(%)	(%)	(%)
1	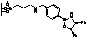	**94**	40	**91**	58	**90**	64	**90**	71	**90**	75	**90**	79
	S1-3Br												
2	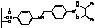	**68**	50	**68**	57	**68**	64	**68**	67	**68**	72	**68**	75
	S1-Ph3Br												
3	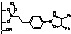	**90**	46	**90**	50	**90**	57	**90**	67	**90**	73	**90**	81
	S3-2												
4	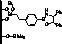	**90**	60	**90**	74	**90**	85	**90**	88	**90**	90	**90**	90
	S3-2p												
5	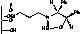	**72**	72	**72**	75	**72**	80	**72**	80	**72**	80	**72**	80
	S2-3Br												
6	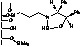	**72**	82	**72**	92	**72**	92	**72**	92	**72**	92	**72**	92
	S2-3pBr												
7	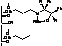	**72**	33	**72**	40	**72**	49	**72**	58	**72**	64	**72**	64
	S2-3dI												

Oxazaborolidines are known to be air moisture sensitive in homogeneous phase and this is also the case for the supported materials. Indeed, when the supported material is deliberately removed from an inert atmosphere, a complete loss of enantiomeric excess is observed (Table 10, cycle 7). Nevertheless, its enantioselectivity can be partially recovered when the residual physisorbed water is eliminated by azeotropic distillation with toluene via a Dean Stark apparatus (Table 10, cycle 8).

**Table 10:** Regeneration of the supported oxazaborolidine.

Supported oxazaborolidine	Cycle 1		Cycle 6		Cycle 7^a^		Cycle 8^b^	
	ee	Conv	ee	Conv	ee	Conv	ee	Conv
	(%)	(%)	(%)	(%)	(%)	(%)	(%)	(%)
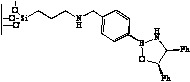	**94**	40	**90**	90	**0**	46	**63**	40
S1-3Br								

a) in the presence of air; b) regeneration via a Dean Stark apparatus with anhydrous toluene for 24 h.

## 3. Experimental

### 3.1. General

All the substrates were purchased from Aldrich and used as received. THF was distilled under argon on sodium/benzophenone. Toluene was distilled under argon on sodium. All the experiments were carried under argon atmospheres with Schlenk techniques. The GC column is a Restek Rt-βDEXsm chiral column, length 30 m, diameter 0.25 mm. 

### 3.2. Preparation of the homogeneous catalysts

*(4S,5R)-4,5-Diphenyl-1,3,2-oxazaborolidine* (Table 3, entry 1). (1*S*,2*R*)-(+)-2-Amino-1,2-diphenyl-ethanol (1 g, 4.69 mmol) is dissolved in anhydrous THF (10 mL), then a solution of borane in THF (9.38 mL, 1 M, 9.38 mmol) is added at -78 ºC for 30 min. The resulting solution is gradually warmed to 30 ºC and stirred at 30 ºC during 10 h. The excess of borane is eliminated under vacuum. IR (KBr), 3,070, 3,028, 2,931, 2,867, 1,599, 1,452 cm^-1^, ^1^H-NMR (250 MHz, CDCl_3_) δ ppm, 7.40–7.29 (m, 10 H, Ar-H), 5.13–5.11 (d, 1 H, C-H), 4.39–4.37 (d, 1H, C-H), 2.18–2.12 (s, 1 H, N-H), yield 100%. See procedure described by Itsuno *et al*. [1,8].

*(4S,5R)-2,4,5-Triphenyl-1,3,2-oxazaborolidine* (Table 3, entry 2). (1*S*,2*R*)-(+)-2-Amino-1,2-diphenyl-ethanol (1 g, 4.69 mmol) and phenylboronic acid (4.69 mmol) are combined with anhydrous toluene (40 mL) and water is removed using a Dean-Stark apparatus for 24 h. The solvent is removed under vacuum to provide the oxazaborolidine as a colorless oil. IR (KBr), 3,070, 3,028, 2,931, 2,867, 1,599, 1,452 cm^-1^, ^1^H-NMR(250 MHz, CDCl_3_) δ ppm, 7.99–7.96 (d, 1 H, Ar-H), 7.40–7.30 (m, 3 H, Ar-H), 7.20–7.10 (m, 9 H, Ar-H), 4.79–4.76 (d, 7Hz, 1 H, C-H), 4.12–4.10 (d, 7Hz, 1 H, C-H), 2.18–2.12 (s, 1 H, N-H), yield 100%. See procedure described by Quallich *et al*. [3].

*(4S,5R)-2-(4-Butylphenyl)-4,5-diphenyl-1,3,2-oxazaborolidine* (Table 3, entry 3). See procedure described for (4S,5R)-2,4,5-triphenyl-1,3,2-oxazaborolidine. IR (KBr), 3,070, 3,028, 2,931, 2,867, 1,599, 1,452 cm^-1^, ^1^H-NMR (250 MHz, CDCl_3_) δ ppm, 7.96–7.95 (d, 1 H, Ar-H), 7.60–7.50 (d, 1 H, Ar-H), 7.30–7.10 (m, 11 H, Ar-H), 7.10–6.90 (d, 1 H, Ar-H), 4.77–4.74 (d, 7Hz, 1 H, C-H), 4.13–4.11 (d, 7Hz, 1 H, C-H), 2.61–2.55 (t, 7Hz, 2 H, C-H), 2.10–2.08 (s, 1 H, N-H), 1.60–1.50 (m, 7Hz, 2 H, C-H), 1.35–1.25 (m, 7.5 Hz, 2 H, C-H), 0.89–0.84 (t, 8Hz, 3 H, C-H), 2.18–2.12 (s, 1 H, N-H). 

*(4S,5R)-4,5-Diphenyl-2-(4-(2-(triethoxysilyl)ethyl)phenyl)-1,3,2-oxazaborolidine* (Table 3, entry 4). 4-vinylphenylboronic acid (1 g, 6.75 mmol) is dissolved in anhydrous ethanol (20 mL) then triethoxysilane (1.66 g, 10.1 mmol) and Karsted’s catalyst (0.1 equiv. with respect to the complex) is added. The solution color changed to yellow and it was heated at 50 ºC for 12 h, at which point the solution becomes brown. The oxazaborolidine were prepared using the same procedure as described for (4*S*,5*R*)-2,4,5-triphenyl-1,3,2-oxazaborolidine. IR (KBr), 3,070, 3,028, 2,931, 2,867, 1,599, 1,452 cm^-1^, ^1^H-NMR (250 MHz, CDCl_3_) δ ppm, 7.99–7.96 (d, 2 H, Ar-H), 7.74-7.55 (m, 3 H, Ar-H), 7.20–7.10 (m, 9 H, Ar-H), 5.23–5.20 (d, 7 Hz, 1H, C-H), 4.79–4.70 (d, 7 Hz, 1 H, C-H), 3.85–3.70 (q, 8 Hz, 6 H, C-H), 2.63–2.57 (t, 7 Hz, 2 H, C-H), 1.99–1.98 (s, 1 H, N-H), 1.20–1.15 (t, 8 Hz, 9 H, C-H), 0.92–0.88 (t, 7 Hz, 2H, C-H). 

* (4S,5R)-4,5-Diphenyl-3-propyl-1,3,2-oxazaborolidine* (Table 4, entry 2). 1-bromopropane (0.123 mL, 1.35 mmol), (1*R*,2*S*)-2-amino-1,2-diphenylethanol (0.29 g, 1.35 mmol) and triethylamine (0.63 mL, 4.5 mmol) are mixed and heated at reflux during for 18 h. After cooling at 28 ºC, distilled water is added until neutral pH, then the organic phase is recovered with ethyl acetate without further purification. The product is then analyzed by GC/MS. ^1^H-NMR (250 MHz, CDCl_3_) δ ppm, 7.40–7.29 (m, 10 H), 5.12 (d, 8 Hz, 1 H), 4.37 (d, 8 Hz, 1 H), 3.6 (s, 1 H), 2.55 (t, 7 Hz, 2 H), 1.45 (q, 7 Hz, 2 H), 0.9 (t, 7 Hz, 3 H).

### 3.3. Preparation of the heterogeneous catalyst

#### 3.3.1. Functionalization of the Grace silica (S1-1)

Grace Davison silica (1 g) was activated at 150 ºC under vacuum for 3 h. After cooling the silica under argon, anhydrous toluene (40 mL) and 3-aminopropyltriethoxysilane (0.89 g, 4 mmol) is added and the mixture heated at 130 ºC for 12 h; the ethanol formed during the reaction is removed by the Dean-Stark method. The solid is filtered with toluene (3 × 20 mL), methanol (3 × 20 mL), dichloromethane (3 × 20 mL), diethyl ether (3 × 20 mL), then washed in a Soxhlet apparatus for 24 h (dichloromethane/diethylether = 1/1). Finally the solid is dried at 70 ºC for 3 h.

#### 3.3.2. Immobilization of the boronic acid (S1-2Br)

S1-1 (1 g) was activated at 100 ºC under vacuum for 3 h. After cooling the silica under argon, boronic acid (3 mmol) dissolved in anhydrous toluene (40 mL) and triethylamine (1.01 g, 10 mmol) are added and the mixture then heated at 120 ºC for 36 h. The solid is filtered with toluene (3 × 20 mL), dichloromethane (3 × 20 mL), diethyl ether (3 × 20 mL), then washed with a Soxhlet apparatus for 24 h (dichloromethane/diethyl ether = 1/1). Finally the solid is dried at 70 ºC for 3 h (S1-2Cl or S1-2Br).

#### 3.3.3. Formation of the oxazaborolidine (S1-3Br)

S1-2Br (1 g) was activated at 100 ºC under vacuum for 3 h. After cooling the silica under argon, anhydrous toluene (20 mL) is added. (1*S*,2*R*)-(+)-2-Amino-1,2-diphenylethanol (0.288 g, 1.35 mmol) was suspended in anhydrous toluene (40 mL) and heated to 80 ºC to afford a colourless solution. The solution is added to the silica solution then heated at 130 ºC for 5 days, with the water formed being removed by the Dean-Stark method. The solid is then filtered under argon with anhydrous toluene at 80 ºC (3 × 20 mL) and anhydrous THF (3 × 20 mL) (S1-3Br).

#### 3.3.4. Functionalization of the Grace silica (S2-1X)

Grace silica (1 g) was activated at 150 ºC under vacuum for 3 h. After cooling the silica under argon, anhydrous toluene (40 mL) and 3-halogenopropyltriethoxysilane (0.89 g, 4 mmol) are added and the mixture heated at 130 ºC for 12 h, with the ethanol formed during the reaction being removed with a Dean-Stark apparatus. The solid is filtered with toluene (3 × 20 mL), methanol (3 × 20 mL), dichloromethane (3 × 20 mL), diethyl ether (3 × 20 mL), then washed with a Soxhlet apparatus for 24 h (dichloromethane/diethyl ether = 1/1). Finally the solid is dried at 70 ºC for 3 h (S2-1X).

#### 3.3.5. Passivation step of residual silanols

S2-1X (1 g) was activated at 100 ºC under vacuum for 3 h. After cooling the silica under argon, anhydrous toluene (40 mL) and *N,O*-bis(trimethylsilyl)trifluoroacetamide (0.57 mL, 3 mmol) are added and the mixture heated at 80 ºC for 3 h. Then the solid is filtered with toluene (3 × 20 mL), methanol (3 × 20 mL), dichloromethane (3 × 20 mL), diethyl ether (3 × 20 mL), and washed with a Soxhlet apparatus for 24 h (dichloromethane/diethyl ether = 1/1). Finally the solid is dried at 70 ºC for 3 h (S2-1pX).

#### 3.3.6. Immobilization of chiral amino alcohol

S2-1Br (1 g, 0.92 mmol) was activated at 100 ºC under vacuum for 3 h. After cooling the silica under argon, anhydrous toluene (30 mL), (1*R*,2*S*)-2-amino-1,2-diphenylethanol (0.29 g, 1.38 mmol) and triethylamine (0.64 mL, 4.6 mmol) are added and the mixture heated at reflux during 72 h. The solid is filtered with toluene (3 × 20 mL), methanol (3 × 20 mL), dichloromethane (3 × 20 mL), diethyl ether (3 × 20 mL), then washed with a Soxhlet apparatus for 24 h (dichloromethane/diethyl ether = 1/1). Finally the solid is dried at 70 ºC for 3 h (S2-1pX).

#### 3.3.7. Oxazaborolidine formation

S2-2Br (1g, 0.72 mmol) was activated at 100 ºC under vacuum for 3 h. After cooling the silica under argon, anhydrous THF (15 mL) and 1M BH_3_·THF solution (1.44 mL, 1.44 mmol) is added to the solid and allowed to react at -78 ºC for 30 min then heated to 28 ºC for 12 h. Then the excess of borane and the solvent are eliminated under vacuum at 85 ºC for 1 h. 

### 3.4. Catalysis tests

S2-3I (0.5 g, 0.65 mmol/g, 0.325 mmol) is activated during 3 h at 100 ºC under vacuum, then put under argon. 1M BH_3_·THF solution (0.65 mL, 0.65 mmol) is added to the solid and allowed to react at 40 ºC for 20 min then cooled to 28 ºC. 0.1 mL of acetophenone (3.25 mmol) diluted in THF (3 mL) is added dropwise during 30 minutes. Catalytic solution is sampled prior to analysis. Hydrolysis is performed by addition of HCl (2M) to the analytical sample (0.5 mL). The organic phase is extracted with ethyl acetate, washed with a NaOH solution, dried on MgSO_4_ and filtered. The sample is analysed by GC with dodecane as standard (Restek Rt-βDEXsm chiral column, length 30 m, diameter 0.25 mm). The solid catalyst is washed three times with anhydrous THF in order to recover physisorbed product. This way the material is reactivated for the next catalytic cycle.

## 4. Conclusions

Immobilization of oxazaborolidines on mesoporous silica via their boron and nitrogen substituents has been successfully achieved. In order to obtain a high density of active sites, several parameters have been studied: reaction time, nature of the boronic acid, nature of the alkoxysilane and synthesis strategies. Thus, for chiral ligand linked via a boron substituent, the best strategy to reach high density is obtained via the direct functionalization of silica with a boronic acid alkoxysilane (0.85 mmol/g). Regarding immobilization via the nitrogen substituent, the use of a bromo- or iodoalkoxysilane is more suitable (up to 0.72 mmol/g). The silanol passivation step is beneficial for the restriction of physisorbed amino alcohol and consequently, the conversion for the reduction of acetophenone is improved. Moderate to high ee (up to 95%) are obtained with supported oxazaborolidines. Although the presence of a substituent linked to nitrogen or boron element of the chiral ligand leads to a decrease of ee, a higher ee than its equivalent in homogeneous phase is obtained as the result of a better isolation of the active site induced by its immobilization. Finally, no loss of the ee has been observed with any of the supported oxazaborolidines after five reuses. 
